# Bacteriophage Therapy for Clinical Biofilm Infections: Parameters That Influence Treatment Protocols and Current Treatment Approaches

**DOI:** 10.3390/antibiotics9110799

**Published:** 2020-11-12

**Authors:** James B. Doub

**Affiliations:** Division of Infectious Diseases, University of Maryland School of Medicine, Baltimore, MD 21201, USA; jdoub@ihv.umaryland.edu

**Keywords:** biofilm, bacteriophage therapy, prosthesis related infections, hardware infections, left ventricular assist devices

## Abstract

Biofilm infections are extremely difficult to treat, which is secondary to the inability of conventional antibiotics to eradicate biofilms. Consequently, current definitive treatment of biofilm infections requires complete removal of the infected hardware. This causes significant morbidity and mortality to patients and therefore novel therapeutics are needed to cure these infections without removal of the infected hardware. Bacteriophages have intrinsic properties that could be advantageous in the treatment of clinical biofilm infections, but limited knowledge is known about the proper use of bacteriophage therapy in vivo. Currently titers and duration of bacteriophage therapy are the main parameters that are evaluated when devising bacteriophage protocols. Herein, several other important parameters are discussed which if standardized could allow for more effective and reproducible treatment protocols to be formulated. In addition, these parameters are correlated with the current clinical approaches being evaluated in the treatment of clinical biofilm infections.

## 1. Introduction

**Figure 1:**
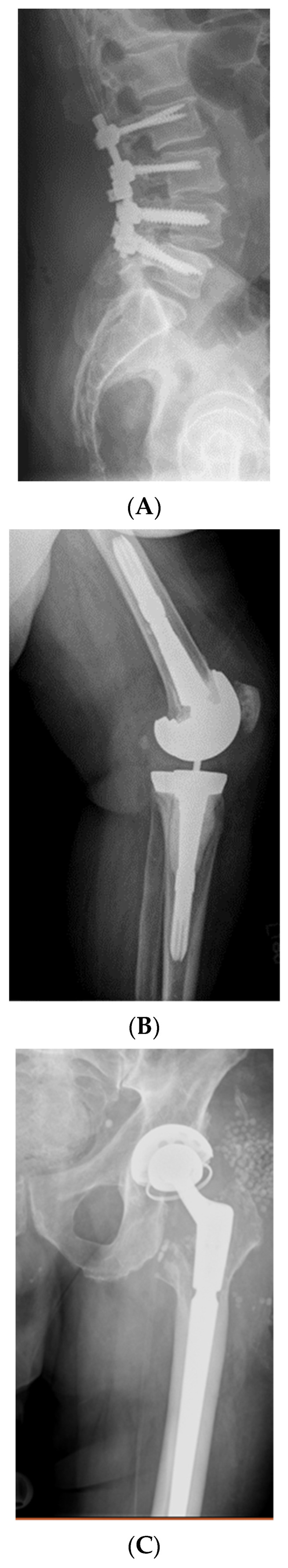
Examples of a few types of “hardware” that once infected require removal for definitive cure of these biofilm infections. (**A**) A lumbar posterior spinal rods and pedicle screw construct. (**B**) Total knee arthroplasty with long stem femoral and tibial components. (**C**) Total hip arthroplasty.

When bacteria attach to surfaces they can form an extracellular matrix comprised of proteins, polysaccharides, extracellular DNA and water [1,2,3,4,5]. The extracellular matrix and the bacteria that reside within this matrix are what comprise biofilms. Contrary to planktonic bacteria that are free floating, biofilm bacteria are sessile. Bacteria in these sessile states have drastically different characteristics than planktonic bacteria causing conventional antibiotics to have limited ability to eradicate biofilms [1,2,3,4,5]. This stems from the reduced metabolic activity of biofilm bacteria and the architecture of biofilm itself [1]. The minimal inhibitory concentration of antibiotics to biofilm bacteria can be up to 1000 times that of planktonic bacteria [1]. Therefore to definitively cure these infections surgical removal of all the hardware (Figure 1) that harbor biofilms, in combination with prolonged systemic antibiotic therapy, is required. However, this causes significant morbidity and mortality to the patients who suffer from these infections. As a result, new antimicrobial methods are needed that can treat these biofilm infections without removal of the hardware. Bacteriophages might be such an adjuvant therapeutic.

### 1.1. Bacteriophages

Bacteriophages are viruses with a very narrow spectrum of activity to only certain strains of a certain bacterial species. Infection of human cells has not been observed and therefore bacteriophages are attractive therapeutics to use in bacterial infections [6,7]. These viruses can either be lytic or lysogenic. Lytic bacteriophages hold the most promise in treating infections given their ability to lyse bacteria. Lysogenic bacteriophages incorporate into bacterial DNA and do not induce bacterial lysis until reactivated at a later time, making them not advantageous in the treatment of infections. In nature, the majority of bacteria live in sessile states associated with biofilms and through evolution bacteriophages have coevolved to be able to infect and lyse bacteria inside biofilms [6,7].

### 1.2. Bacteriophage Activity in Biofilms

In order to eradicate a clinical biofilm, an effective agent must be able to penetrate the biofilm and kill the bacteria that are present in various metabolic states while also degrading the biofilm extracellular matrix. Bacteriophages possess these abilities, but are not motile agents [6,7]. Therefore if bacteriophages can establish an infection within a biofilm, high rates of replication can occur given the high densities of biofilm bacteria in a structured space [8]. Bacteriophages even retain lytic activity against reduced metabolically active bacteria [9,10]. In the deepest regions of a biofilm, bacteria known as persister cells are semi-dormant [11]. All conventional therapeutics have limited activity to persister cells [11]. However, bacteriophages have the ability to infect persister cells and then lyse these bacteria once they become metabolically active again [12]. 

Bacteriophages also can enzymatically degrade the biofilm extracellular matrix thus allowing for dissemination within the biofilm. This occurs through use of endolysins and depolymerases [13,14]. Enodolysins are enzymes produced by bacteriophages to weaken the bacterial cell wall allowing for lysis to occur releasing their progeny [13]. Endolysins also have activity in degrading the extracellular matrix [13]. Depolymerases are enzymes attached to some bacteriophages that can also degrade the biofilm matrix in functionally different ways to endolysins [13]. Unique to bacteriophages is their ability to self-replicate and increase their own concentrations. This occurs when bacteriophage induced bacterial lysis causes release of progeny into the local environment to infect other bacterial cells. In the confined space of a biofilm this could be advantageous allowing for bacteriophages to infect biofilm bacteria and slowly degrade the biofilm [6]. However bacteriophages are not motile agents and finding biofilm bacteria may be an arduous undertaking if not directly applied to the biofilm. 

Several preclinical animal studies support the use of bacteriophage therapy in clinical biofilm infections [15,16,17,18,19,20,21,22,23]. These studies show that local administrations of bacteriophages to the site of biofilm infections result in biofilm reduction [15,16,17,18,19,20,21,22,23]. In addition, these studies show that, without local administration of bacteriophage therapy, reduction in biofilms on hardware is not significantly reduced [19]. One of the most relevant preclinical studies was conducted by Morris et al. [19]. Rats were implanted with replica orthopedic prosthetics and then infected with *Staphylococcus aureus.* A total of 3 weeks later rats were given intraperitoneal bacteriophage therapy for 3 days. Results show synergistic activity of bacteriophage therapy with vancomycin in local infected tissues but no statistical reductions in biofilm burden on infected prosthetic material [19]. These findings support other preclinical testing that direct instilment of bacteriophage therapy to the site of biofilm infection is needed to achieve significant biofilm reduction. The intrinsic abilities of bacteriophages and results of animal studies support evaluation of bacteriophages in the treatment of clinical biofilm infections. However bacteriophages are not like conventional antibiotics and several parameters need to be understood before using this therapeutic in vivo. 

## 2. Parameters that Impact Treatment Protocols

Unlike conventional antibiotics, bacteriophage therapy is not a one size fits all antimicrobial therapeutic. Rather a bacteriophage that has robust activity to a clinical isolate of a specific bacterial species may have widely different activity or no activity to another clinical isolate of the same bacterial species. Many other aspects of bacteriophage therapy are poorly understood and not standardized, making creation of treatment protocols an arduous undertaking. At the present time, standardization of protocols can only be achieved with respect to bacteriophage titers and duration of therapy. However, this limits bacteriophage therapy to be used similarly to conventional antibiotics and does not incorporate many other parameters that need to be considered to devise advantageous, reproducible treatment protocols. Herein several additional important parameters are discussed.

### 2.1. Current “Susceptibility” Testing

At the present time, bacteriophage therapy requires a clinical isolate to be tested against either a library of individual bacteriophages or to a set cocktail of bacteriophages to ensure susceptibility. Given the narrow spectrum of activity, even with the use of bacteriophage cocktails, susceptibility testing is warranted. There is no proverbial gold standard of susceptibility testing and no standardized “breakpoints” are available to determine if a bacteriophage has adequate activity to be used clinically. Therefore, it is vital to understand how in vitro susceptibility testing is conducted to be able to extrapolate these findings to in vivo use. Testing for phage susceptibility usually includes two methods.

**Figure 2:**
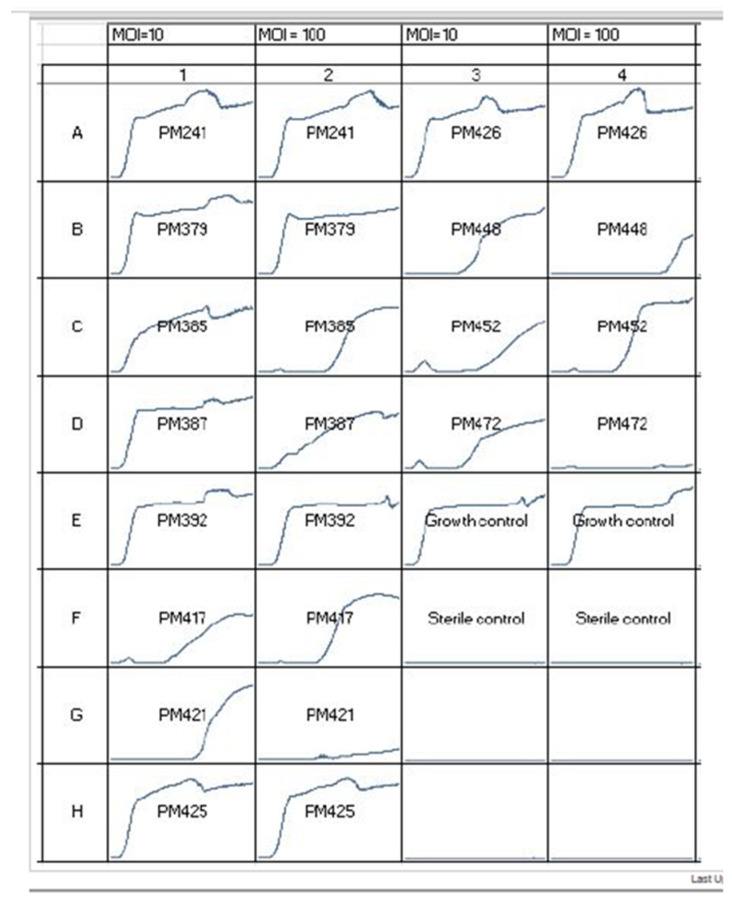
Bacterial growth inhibition curves or “Phagogram” for a compassionate use *Staphylococcus epidermidis* case in a recalcitrant prosthetic joint infection. Different bacteriophages are indicated by PM241-PM472. MOI refers to multiplicity of infection. Growth control is the bacterial isolate with no bacteriophages. Each box has time on the *x*-axis from 0 to 48 h. This figure shows how growth inhibition testing is conducted to determine potential bacteriophage candidates (PM448, PM472, and PM421). This figure also shows how different MOIs can cause different growth inhibition durations as seen with bacteriophages: PM448, PM472, and PM421.

(1) Bacterial growth inhibition or “Phagogram”: This is conducted when a clinical bacterial isolate is grown in vitro and then inoculated into wells of a 96-microwell plate. The concentration of bacteria in each well is standardized. Then several different bacteriophages (or cocktails) that have potential activity are applied to the wells and monitored for 24 to 48 h to compare growth inhibition to positive controls (Figure 2). It should be noted that the multiplicity of infection (MOI) is usually 100:1. This means bacteriophages outnumber the bacteria 100 to 1. Bacteriophages that inhibit growth of bacteria for durations longer then the positive control are considered candidates. However, no standardized time durations have been established for what is considered long enough growth inhibition to be used in vivo.
(2) Formation of plaques: Once candidate bacteriophages are determined based on growth inhibition, the ability to form plaques on lawns of the bacterial isolate are then conducted. This usually is conducted with double agar overlay plaque assays.

Bacteriophages that form plaques and can inhibit bacterial growth are considered potential therapeutic options. Complicating this testing is that different MOIs can have drastically different growth inhibition durations. For instance an MOI of 100 might inhibit growth for 24 h while an MOI of 10 for the same bacteriophage might not inhibit growth at all. Figure 1 reinforces this for a *Staphylococcus epidermidis* clinical isolate in which PM448, PM472, PM421 have different growth inhibition durations for different MOIs of 100 and 10. This has ramifications when treating biofilm infections as reproducing the high MOI seen in vitro may not occur unless direct bacteriophage application is applied to biofilms. This can also have implications for resistance formation which will be discussed below.

Another important implication of this testing is the lack of standardization with respect to the duration of growth inhibition. A bacteriophage that inhibits growth for 48 h likely has different therapeutic potential compared to a different bacteriophage that only inhibits growth for 8 h. In correlation, different in vivo bacterial metabolic states may require different levels of growth inhibition. For instance, biofilm bacteria are less metabolically active then planktonic bacteria and therefore less in vitro growth inhibition might be needed compared to if bacteriophage therapy is being used to treat a planktonic infection. No standardized growth inhibition duration has been proposed, thereby exposing treatment protocols to potential reproducibility issues. To improve reproducibility, it may be important to standardize what is considered adequate growth inhibition depending on how a bacteriophage therapy is going to be used (intravenously vs. directly applied to biofilms). It should also be mentioned that “susceptibility” testing is usually only conducted against planktonic bacteria. Routine testing for a bacteriophage’s ability to remove in vitro biofilms is usually not conducted. However, in the treatment of biofilm infections, determining the ability of a candidate bacteriophage (or cocktail) to reduce in vitro biofilms should be considered as an additional susceptibility testing step once adequate growth inhibition and formation of plaques has been proven.

### 2.2. Pharmacology

The main routes of phage administration that are being investigated in western medicine for the treatment of biofilm infections are local administration directly applied to the infected hardware and intravenous therapy. Eastern European studies have had limited success with topical or oral phage therapies in the treatment of biofilm infections and therefore little interest is present for these methods beyond topical application for burns and wounds [24,25,26]. Given the novelty of this therapeutic there is a paucity of data with respect to pharmacokinetics of local administration of bacteriophage therapy to biofilms. No data are present to suggest how long locally administrated bacteriophage reside at the infection site, how much is systemically absorbed or the safety of this approach. On the other hand, intravenous bacteriophage therapy has been more widely used and data are present to help guide treatment protocols. Therefore discussion about simple pharmacokinetics is limited to intravenous use.

Distribution: Bacteriophages are expected to be diluted in the whole body volume when given intravenously [27]. In numerous animal studies the titers of bacteriophages after intravenous infusions can be reduced 100–100,000-fold within 30 min of infusion [27]. Animal studies have shown distribution to various other organs including but not limited to heart, lungs, brain, skeletal muscle, bone marrow, and genitourinary tract [27]. However there are no data on intravenous bacteriophage therapy distribution to joints, spinal hardware, Left ventricular assist devices (LVADs) or other spaces that could have poor vascularization.

Metabolism: This is the chemical modification of bacteriophage therapy to reduce its activity. With bacteriophage therapy this occurs mainly through inactivation by the human immune system by neutralizing antibodies [28]. Neutralizing antibody responses have occurred with all forms of bacteriophage administration and this is a theoretical concern for long duration bacteriophage therapies [28,29].

Elimination: Elimination occurs mainly through hepatic clearance. In 1969, using a T4 bacteriophage, Inchley demonstrated that the liver phagocytosed and eliminated more than 99% of the bacteriophages within 30 min after systemic administration [30]. Other studies have supported this extensive hepatic elimination [30,31,32,33]. In one compassionate use case, 50 min after intravenous administration no bacteriophage could be detected in patient’s serum [34].

Based on these data, intravenous bacteriophage therapies are likely to have significant reduction in titers, secondary to volume of distribution and hepatic elimination. Therefore, achieving MOIs similar to what occurs with in vitro susceptibility testing may be difficult. With the use of bacteriophage therapy applied directly to biofilm infections, MOIs may be similar to what was observed with in vitro susceptibility testing. However, limited pharmacological data have been found to help direct dosing, duration or safety of local bacteriophage administration.

### 2.3. Safety

Unbeknownst to most, humans are exposed to low titers of bacteriophages on a continual basis [35]. Eastern European medicine has used bacteriophage therapy for close to 100 years with few significant adverse reactions being reported [36]. However, given the extensive hepatic clearance, western medicine is entertaining the use of high titers (greater than 10^9^) of intravenous bacteriophage therapy and direct injection of high titers of bacteriophages directly to biofilm infections. Limited safety studies have been conducted using these techniques beyond a phase 1 clinical trial evaluating a three-bacteriophage cocktail with titers of 1 × 10^9^ plaque-forming unit (PFU) twice a day for 14 days to *Staphylococcus aureus* bacteremia [37].

While intravenous bacteriophage therapy has been used in the past with limited adverse events, recent compassionate use cases have shown two adverse events [38,39]. One occurred in the treatment of chronic pseudomonas left ventricular assist device (LVAD) infection in which no success occurred with low titers of intravenous bacteriophage therapy and subsequent bacteriophage therapy with high titers of 1 × 10^11^ PFU induced fever, shortness of breath and wheezing [38]. These symptoms resolved with supportive medical care but continued with repeat dosing with the same titers. When titers were diluted to 1 × 10^10^ PFU, the authors document that no adverse events occurred [38]. Endotoxin units were well below the United States Food and Drug Administration approved limit. In the other case, a significant transaminitis occurred after three doses of daily intravenous bacteriophage therapy with titers of 2.7 × 10^9^ PFU in the treatment of a recalcitrant methicillin-resistant *Staphylococcus aureus* prosthetic joint infection. No causative etiology other than bacteriophage therapy could be found [39]. After cessation of bacteriophage therapy liver function returned to normal after 14 days. These two cases suggest that an upper limit may exist with respect to the titers that can be intravenously infused without exposing patients to potential adverse events. However only further safety trials with high titers given intravenously or directly to sites of biofilm infections will be able to assess safety and if there is a ceiling for the amount of titers that can be given.

### 2.4. Resistance Development

With longer bacteriophage therapies, concern arises for the development of resistance. Resistance usually occurs from bacterial modifications of cell surface receptors or down regulation of receptors used in phage–bacteria attachment [40,41]. Other means of resistance can occur through adaptive systems such as the CRISPR–Cas9 system that cleaves phage DNA thus not allowing for progeny phage to be created [40]. Means to overcome or prevent resistance from occurring include use of cocktails of bacteriophages and bacteriophage substitutions. Bacteriophage cocktails are a group of different bacteriophages that theoretically use different attachment receptors. Therefore, if resistance develops to one receptor, the cocktail should continue to be effective. A recent study showed the frequency of spontaneous induction of resistance to a cocktail of three *Staphylococcus aureus* bacteriophages was no greater than 3 × 10^−9^ [42]. Bacteriophage substitutions are simply changing therapy to a different bacteriophage that has lytic activity to the bacterium.

Bacteriophage resistance can occur rapidly causing formation of resistant variants that are immune to further bacteriophage infection [41]. This could impede effectiveness of bacteriophage therapy but resistance may also come at a cost to the bacterium especially when antimicrobial agents are present [41]. Moreover, bacteriophage-resistant bacteria often lack important surface features that are responsible for bacterial virulence [41]. Nonetheless resistance is an important factor that should be accounted for especially with prolonged bacteriophage treatments. In a case series of 10 intravenous bacteriophage only cases, resistance occurred in a significant portion of patients and required bacteriophage substitutions [38]. Resistance development is dependent on complex interplays of MOIs, growth inhibition durations and other bacteriophage–bacteria interactions [41]. Therefore resistance might develop rapidly or slower depending on these complex interactions. Determining in vitro resistance development to a clinical isolate is not routinely conducted, but could be easily assessed with susceptibility testing by evaluating the bacterial overgrowth for resistant variants. Resistance development is an important parameter that can have ramifications on efficacy and reproducibility of treatment protocols. Therefore it might be prudent to routinely test for and standardize what an acceptable level of in vitro resistance development is for different infectious processes to reduce further problems of reproducibility and improve efficacy of treatment protocols.

### 2.5. Synergistic or Antagonistic Activity with Antibiotics

While resistance is an important parameter so is compatibility with systemic antibiotics which may have synergistic or antagonistic activity with bacteriophage therapy. Theoretically, antibiotics that inhibit protein synthesis (rifampin, tetracyclines, linezolid and others) can inhibit phage gene expression and therefore be antagonistic [43]. Antibiotics that inhibit cell wall synthesis inhibitors such as beta-lactams are potentially more synergistic [43]. These findings have been reinforced in numerous in vitro studies [43]. It has also been documented that concentrations of antibiotics also have important ramification of synergistic or antagonistic activity with higher antibiotic concentrations tending to be more antagonistic compared to lower concentrations which tend to be more synergistic [43]. It is interesting that in vivo studies have shown more synergistic activity of antibiotics with bacteriophage therapy then antagonism [15,16,17,18,19,20,21,22,23]. Spatial and temporal interactions of antibiotics and bacteriophages in vivo likely account for this synergistic activity [43]. It should be reinforced that biofilm bacteria reside where there is poor vascularization and therefore very low concentrations of systemic antibiotics may make phage–antibiotic compatibility less of an issue in vivo [43]. Testing for in vitro for phage–antibiotic compatibility is not commonly conducted. As with resistance development and susceptibility testing, it may be prudent to ensure bacteriophage–antibiotic compatibility with the systemic antibiotics that are planned to be used thus allowing for more reproducible treatment protocols.

### 2.6. Clinical Biofilms

In vitro bacteriophage biofilm studies are traditionally conducted in static environments. These studies are usually devoid of human plasma proteins, lack in vivo stressors and normally remove planktonic infections before experiments are conducted. However, in vivo, planktonic infections overly clinical biofilm infections and are what causes the majority of the symptoms that patient’s experience. Without eradicating these planktonic bacteria, bacteriophage therapy protocols will have to account for the planktonic infection and the biofilm infection. This adds complexity to the use of bacteriophage therapy and potentially further hinders reproducibility given the heterogeneity of these planktonic infections.

Other clinical factors that should be considered include: stability of infected hardware and importance of manual debridement of biofilms. Stability of hardware must be assessed to ensure retaining these materials is possible. This is usually conducted by imaging but manual inspection and manipulation is the most advantageous way to assess the ability to retain these materials. Manual debridement of biofilms has also been shown to be synergistic with respect to bacteriophage activity in biofilms [16,17]. This synergistic activity is likely a result of better bacteriophage penetration into biofilm and exposing biofilm bacteria to bacteriophages [16,17].

### 2.7. Conclusions

The parameters discussed show that currently bacteriophage therapy is not a therapeutic that can be used similarly to conventional antibiotics. In addition relying mainly on bacteriophage’s ability to self-replicate is unlikely to be beneficial beyond isolated case reports given the complexity of these other parameters. Rather thoughtful consideration of many parameters needs to be considered to devise effective, reproducible treatment protocols. Most of these parameters discussed are intertwined but standardization is lacking. Given the heterogeneity of these parameters glaring issues of reproducibility are present at this nascent stage of bacteriophage therapy. To reduce these reproducibility issues standardizing some of these parameters might be needed which include: minimal duration of growth inhibition, resistance testing, bacteriophage–antibiotic compatibility and ensuring in vitro bacteriophage biofilm activity. This may allow for more rigorous testing of reproducible protocols to therefore definitively determine if this therapeutic has efficacy in treating clinical biofilm infections.

## 3. Current Theoretical Bacteriophage Protocols for Chronic Biofilm Infections

Many recent compassionate use cases have been conducted recently to treat clinical biofilm infections (prosthetic joint infections, LVAD infections, vascular graft infections and others). Two main approaches are being used in western medicine which include: intravenous bacteriophage therapy and the use of surgical interventions to directly inject bacteriophages to site of the biofilm. Table 1 discusses the advantages and disadvantages to each approach in relation to the parameters discussed above. Both approaches involve adjuvant bacteriophage therapy in combination with standard of care systemic antibiotics. Further review of recent case reports with respect to the different approaches is discussed here.

### 3.1. Case Studies of Intravenous Bacteriophage Therapy in Biofilm Infections

Intravenous bacteriophage therapy has been attempted to treat prosthetic joint infections, ventricular assist devices, vascular graft infections and other hardware infections [38,44,45,46]. In one case series, the authors describe the use of bacteriophage therapy for two *Pseudomonas* LVAD infections with prolonged intravenous bacteriophage therapies with unsuccessful outcomes [38]. The same author also treated a *Staphylococcus aureus* LVAD infection with intravenous bacteriophage therapy and the was documented as a success, but the patient continued to have culture positive *Staphylococcus aureus* infection at the time of LVAD explant, suggesting the inability of intravenous bacteriophage therapy to eradicate the biofilm infection [38,44]. Another case report treated a *Klebsiella pneumonia* prosthetic joint infection with 8 weeks of intravenous bacteriophage therapy with improvement of symptoms [45]. However the patient remains on chronic indefinite oral suppression antibiotics, limiting the ability to assess if eradication of the clinical biofilm was achieved.

Intravenous bacteriophage therapy is optically attractive in that no surgery is needed. However at the present time no case report has definitively shown the ability to eradicate clinical biofilms with this approach. The theoretical concern with intravenous bacteriophage therapy alone is the entrapment of bacteriophages in the planktonic infection limiting exposure of bacteriophages to the biofilm bacteria. In correlation, bacteriophages are extensively cleared by the liver and in vivo biofilms are usually poorly vascularized. Therefore achieving theoretical MOIs seen with in vitro “susceptibility” testing requires very high titers of infused bacteriophages. These high titers may be limited by potential adverse reactions [38,39]. Further complicating intravenous only therapies is the need for prolonged durations driven by limited bacteriophages reaching their bacterial biofilm targets thereby leading to the development of resistance and neutralizing antibodies. These variables when combined cause numerous confounding variables that may cause significant issues of reproducibility at this nascent stage of bacteriophage therapy.

### 3.2. Case Studies of Direct Injection of Bacteriophages to Biofilms with Surgical Intervention

The addition of bacteriophage therapy with debridement and irrigation surgeries is the other approach that has been used in several case studies [38,47,48,49,50]. This approach involves injection of high titers of bacteriophage phages directly at the site of the biofilm infection thereby circumventing hepatic clearance. The goal of this approach is to potentially cure these infections without need for removal of the hardware. However the risks of a surgical procedure are present and therefore this approach is optically less desirable.

There have been several compassionate use case reports that have shown successful eradication of biofilm infections which include: chronic orthopedic hardware infections, LVAD infection, vascular graft infection, cardiothoracic surgery infections [38,47,48,49,50]. In these case reports different durations of bacteriophage therapy were used. Some cases only required single doses given at the time of surgery while others used drains to continually instill bacteriophage therapy for 7 to 10 days. All cases described no recurrence of bacterial infections while patients were off antimicrobial therapies thereby showing eradication of bacterial biofilms. However with surgical debridement, successful eradication of bacterial biofilm is confounded by the uncertainty of whether it was bacteriophage therapy that was the reason for clearance or if it was the surgical intervention itself. This questioning occurs because success occurs with these surgical debridement procedures without adjuvant bacteriophage therapy albeit at low rates. In addition, limited data are present to help direct safety, appropriate dosing and durations of directly administered bacteriophage therapies but this approach may allow for more standardized reproducible protocols.

## 4. Conclusions

Many aspects of bacteriophage therapy allow this therapeutic to be an attractive adjuvant therapeutic in the treatment of biofilm infections but much work is needed before definitive efficacy trials are to be conducted. The various parameters discussed here should allow researchers to be more cognizant of the current inherent limitations of bacteriophage therapy. However, given the heterogeneity of these parameters, projected issues of reproducibility are glaring. Therefore, standardizing some of these parameters is warranted to formulate reproducible protocols that will allow for rigorous testing of this therapeutic in the treatment of biofilm infections.

## Figures and Tables

**Table 1:** Advantages and disadvantages of intravenous and direct injection of bacteriophage therapy for clinical biofilm infections.

	Direct Bacteriophage Therapy in Correlation with Surgical Interventions	Intravenous Bacteriophage Therapy
Advantages	Potentially shorter course with less risk of resistance and neutralizing antibodies occurring Direct injection of high titers to biofilm thereby achieving theoretical MOIs similarly to in vitro testing Removes majority of planktonic infection Ensures hardware salvageable Ensures no other pathogens present Allows for manual scrubbing of biofilm	Circumvent surgery and risks of general anesthesia No wounds created that thus no risk for further infections No confounders with proving efficacy either it works or does not work
Disadvantages	Risks of Anesthesia Risks of poor wound healing Chance for introduction of another pathogen during surgical interventions	Have to treat both planktonic and biofilm infection Limited ability to achieve MOIs that were tested with in vitro testing Limited identification of all pathogens involved to match to bacteriophage therapy Unable to assess prosthesis stability beyond radiographic findings Longer therapy with higher risk of resistance and neutralizing antibodies occurring
